# On the potential role of glutamate transport in mental fatigue

**DOI:** 10.1186/1742-2094-1-22

**Published:** 2004-11-04

**Authors:** Lars Rönnbäck, Elisabeth Hansson

**Affiliations:** 1 Institute of Clinical Neuroscience, Göteborg University, Göteborg, Sweden

**Keywords:** Astroglia, microglia, TNF-α, IL-1β, IL-6, extracellular glutamate ([Glu]_ec_), glutamate transport

## Abstract

Mental fatigue, with decreased concentration capacity, is common in neuroinflammatory and neurodegenerative diseases, often appearing prior to other major mental or physical neurological symptoms. Mental fatigue also makes rehabilitation more difficult after a stroke, brain trauma, meningitis or encephalitis. As increased levels of proinflammatory cytokines are reported in these disorders, we wanted to explore whether or not proinflammatory cytokines could induce mental fatigue, and if so, by what mechanisms.

It is well known that proinflammatory cytokines are increased in major depression, "sickness behavior" and sleep deprivation, which are all disorders associated with mental fatigue. Furthermore, an influence by specific proinflammatory cytokines, such as interleukin (IL)-1, on learning and memory capacities has been observed in several experimental systems. As glutamate signaling is crucial for information intake and processing within the brain, and due to the pivotal role for glutamate in brain metabolism, dynamic alterations in glutamate transmission could be of pathophysiological importance in mental fatigue. Based on this literature and observations from our own laboratory and others on the role of astroglial cells in the fine-tuning of glutamate neurotransmission we present the hypothesis that the proinflammatory cytokines tumor necrosis factor-α, IL-1β and IL-6 could be involved in the pathophysiology of mental fatigue through their ability to attenuate the astroglial clearance of extracellular glutamate, their disintegration of the blood brain barrier, and effects on astroglial metabolism and metabolic supply for the neurons, thereby attenuating glutamate transmission. To test whether our hypothesis is valid or not, brain imaging techniques should be applied with the ability to register, over time and with increasing cognitive loading, the extracellular concentrations of glutamate and potassium (K^+^) in humans suffering from mental fatigue. At present, this is not possible for technical reasons. Therefore, more knowledge of neuronal-glial signaling in *in vitro *systems and animal experiments is important.

In summary, we provide a hypothetic explanation for a general neurobiological mechanism, at the cellular level, behind one of our most common symptoms during neuroinflammation and other long-term disorders of brain function. Understanding pathophysiological mechanisms of mental fatigue could result in better treatment.

## Background

Mental fatigue with reduced capacity for attention, concentration, and learning, as well as subsequent disturbance of short-term memory, is a common symptom in diseases with general or patchy neuroinflammation, such as multiple sclerosis (MS) and neurodegenerative diseases, such as Ahlzheimer's and Parkinson's diseases [1-6]. The mental fatigue often appears prior to other more prominent mental, cognitive, or physical symptoms from the nervous system in these diseases. Mental fatigue is also common during the rehabilitation after meningitis or encephalitis (postinfectious mental fatigue), stroke or brain trauma (posttraumatic mental fatigue), being especially troublesome when major neurological symptoms have disappeared and the patient is on his way back to work. According to the International Classification of Diseases, 10th revision (ICD-10), mental fatigue is covered by the diagnoses "mild cognitive disorder" or "neurasthenia" and according to the *Diagnostic and Statistical Manual of Mental Disorders*, 4th edition [7], mental fatigue is included in the group of "mild neurocognitive disorders". According to the diagnostic classification by Lindqvist and Malmgren [8], mental fatigue is one of the symptoms of the "astheno-emotional syndrome".

Although mental fatigue is not exactly the same as depression, where the patient has a feeling of not being able to do anything, there are overlaps and both disorders have behavioral manifestations such as reduction in motivation that would appear similar in animal models, where affective state is either irrelevant or difficult to assess. Even the "sickness behavior" [9] contains a component of fatigue. Mental fatigue is also prominent after sleep deprivation. In addition to the fatigue itself, the patient with mental fatigue often suffers from loudness and light sensitivity, irritability, affect lability, stress intolerance, and headaches [8].

Mental fatigue appears as a decreased ability to intake and process information over time. Mental exhaustion becomes pronounced when cognitive tasks have to be performed for longer time periods with no breaks (cognitive loading). Often, the symptoms are absent or mild in a relaxed and stress-free environment. To explore the possible cellular neurobiology of mental fatigue, we start by looking at some components important for information intake and processing within the central nervous system, namely glutamate neurotransmission, and focus on the clearance of extracellular glutamate ([Glu]_ec_).

## Glutamate neurotransmission is indispensable for information intake and processing within the central nervous system

Glutamate neurotransmission is crucial in information intake and information processing within the brain [see [10]]. Glutamate transmission is also indispensable for long-term potential (LTP) formation, the cellular correlate to memory formation [see [11]].

In brain, the [Glu]_ec _has to be maintained at approximately 1–3 μM in order to assure a high precision (high signal-to-noise ratio) at normal glutamate neurotransmission [12] and also, to avoid excitotoxic actions of glutamate on neurons. The clearance of glutamate from the extracellular space is achieved by high-affinity, sodium (Na^+^)-dependent electrogenic uptake transporters. The glutamate aspartate transporter (GLAST) and glutamate transporter 1 (GLT-1) are most abundantly located on astrocytes surrounding synapses of glutamate-bearing neurons [13]. In fact GLAST and GLT-1 have different expression patterns. GLAST is the major transporter for glutamate uptake during development while expression of GLT-1 increases with the maturation of the nervous system. Glutamate transporter 1 expression seems to follow the formation and maturation of synapses and especially synaptic activity [14]. Even more convincing for the role of astroglia in keeping the [Glu]_ec _low, it has been demonstrated with knockout techniques in rats that loss of GLT-1 or GLAST produces elevated [Glu]_ec _and neurodegeneration characteristic of excitotoxicity, while the loss of neuronal glutamate transporter does not elevate [Glu]_ec _[15].

## Regulation of astroglial glutamate transporter capacity – role of proinflammatory cytokines

A large number of factors have been shown to affect the activity and expression of the glutamate transporters GLT-1 and GLAST. For example, GLT-1 is stimulated by phosphorylation by protein kinase C (PKC), while GLAST is inhibited by PKC at a non-PKC consensus site [16]. The synthesis of GLT-1 has been shown to be stimulated by factors acting via receptor tyrosine kinases and pathways dependent on phosphatidylinositol-3-kinase (PI3K) and the nuclear transcription factor NFκB. One mechanism of regulation of GLT-1 is related to formation of cysteine bridges. Glutamate transporter 1 contains cysteines that are sensitive to oxidative formation of cysteine bridges. Oxidative species such as hydrogen peroxide can readily oxidize the functional sulfhydryl groups of cysteines, to form disulfide bridges which exert an inhibitory effect towards glutamate transports [17]. Examples of factors or altered conditions that impair astroglial glutamate transport are arachidonic acid, lactic acid, cytokines, and leukotrines, nitric oxide (NO), β-amyloid protein, peroxynitrate, and glucocorticoids. The altered conditions could be disturbed energy metabolism with lowering of adenosine triphosphate (ATP) levels or lowering of pH. Notable is the finding that many of these substances or conditions also decrease astroglial gap junction communication and even disintegrate the BBB, thus impairing the astroglial support of the glutamate neurotransmission [for references, see [18]].

Proinflammatory cytokines tumor necrosis factor-α (TNF-α), interleukin (IL)-1β and IL-6 have since long been known to impair astroglial glutamate uptake even if the mechanisms are not fully understood. The inhibitory function of TNF-α was established as early as the 1990s, when TNF-α was shown to inhibit astroglial glutamate uptake [19]. Hu and coworkers [20] reported a dose-dependent inhibition of astrocyte glutamate uptake by a mechanism involving nitric oxide (NO). In a study from 2001, Liao and Chen [21] demonstrated that TNF-α potentiates glutamate-mediated oxidative stress, which results in a decrease in glutamate transporter activity. Recently, Wang and coworkers [22] showed a reduced expression of GLT-1 and GLAST, and also, an impaired glutamate transport in human primary astrocytes, by TNF-α. The nuclear factor NFκB has been suggested to be involved in this regulation [23]. Even IL-1β and IL-6 have been shown to impair astroglial glutamate uptake capacity by involvement of oxidative stress or NO [20,24,25].

Even dysregulation of the blood brain barrier (BBB) is seen early in neuroinflammation, and parallels the release of proinflammatory cytokines [26-28]. Mechanisms for disruption of the BBB in neuroinflammation are incompletely understood, but appear to involve direct effects of cytokines on endothelial regulation of BBB components. Exposure of endothelium to TNF-α interrupts the BBB by disorganizing cell-cell junctions. Furthermore, TNF-α has been shown to depress calcium (Ca^2+^) signaling between BBB endothelial cells by reducing gap junction coupling and inhibiting triggered ATP release [29].

## Could glutamate neurotransmission be dynamically regulated by extracellular glutamate levels?

As stated above, already when the [Glu]_ec _exceeds some 3–5 μM, the efficiency of the glutamate signaling is considered to be reduced [12]. There is prolonged postsynaptic and adjacent glial receptor activation [30], with less precision (with a decreased signal-to-noise ratio) in the glutamatergic transmission. As a consequence, the information taken into the brain will be less distinct. In addition, activation of astroglial networks, with induction of Ca^2+ ^oscillations, both within and between the gap junction-coupled astroglial syncytia [31-33], and with subsequent astroglial glutamate release [34] could increase the excitability level in neighboring neuronal circuits. The overall result may be that more, and larger, neuronal circuits would be activated over time [35,36]. This conclusion is further supported by studies demonstrating that inhibition of GLT-1 could facilitate hippocampal neurotransmission [37] and lead to increased neuronal excitability, as seen in for example hepatic encephalopathy [38].

Increased [Glu]_ec _would also lead to astroglial cell swelling, with a resulting decrease in the extracellular space volume, and locally further increased [Glu]_ec _[39-42]. The astroglial swelling would give rise to relative depolarization of the astroglial cell membrane, with a further decreased astroglial glutamate uptake capacity, and in addition, a decreased capacity of the astrocytes to remove [K^+^]_ec _[43,44]. Even moderately increased (up to 8–10 mM) [K^+^]_ec _levels have been shown in experimental systems to inhibit glutamate release [45].

Recent data indicate a dynamic and fine-tuning regulation of the glutamatergic transmission. One mechanism by which neurons regulate excitatory transmission is by altering the number and composition of glutamate receptors at the postsynaptic plasma membrane. This has been shown for the NMDA receptor in experimental systems and could have prominent importance for dynamic processes as learning and memory [46]. Of great importance in this context are also studies where stimulation of metabotropic glutamate receptors (mGluR3 and mGluR5) have been shown to critically and differentially modulate the expression of glutamate transporters [47] thus creating a substrate for a fine-tuning of the glutamate neurotransmission. Even the proinflammatory cytokine IL-1β could act as a regulator of glutamate transmission, as it was shown recently that this cytokine inhibits glutamate release and reduces LTP as a consequence of the formation of reactive oxygen species [11].

Furthermore, in states of decreased astroglial glutamate uptake capacity, even astroglial glucose uptake, and consequently the supply of metabolic substrates to the neurons, has been reported to decrease [48-50] and there may be relative energy insufficiency at the cellular level in neuronal circuits. In addition, glutamate release from the presynaptic terminals could decrease due to factors such as a decreased glutamine supply of the neurons.

Experimental investigations in the rat and monkey have demonstrated a feedback loop from the left basal frontal cortex, with an inhibitory influence on the locus coeruleus in the brain stem [51]. If this loop also exists in humans, a slight increase in the neuronal firing due to slightly elevated [Glu]_ec _in the basal frontal cortex could lead to a decrease in the noradrenaline and serotonin (5-HT) release in the cerebral cortex, which would also decrease glucogenolysis [52,53] and, furthermore, impair metabolic substrates for cortical neurons.

Thus, it might be that glutamate neurotransmission could be regulated by changing astroglial glutamate transporter capacity, and thus, increases in [Glu]_ec _levels could be one factor to impair glutamate transmission.

## Proinflammatory cytokines and neuroinflammatory and degenerative diseases, major depression, sickness behavior, and sleep deprivation

There is an extensive literature on inflammatory response with microglial activation and the production of proinflammatory cytokines (TNF-α, IL-1β and IL-6) in neuroinflammatory/infectious and neurodegenerative diseases as well as in stroke and trauma [5,54]. The inflammatory activation starts early in some neurodegenerative disease such as Alzheimer's and Parkinson's diseases, being prominent for long time in these diseases and also in neuroinflammatory diseases, in meningitis, encephalitis and in trauma or stroke [see [54]].

Several groups have also described enhanced production of proinflammatory cytokines in major depression [see [55]] and sickness behavior [9,56,57]. This is interesting as there are overlaps between mental fatigue and these disorders. Furthermore, proinflammatory cytokines are activated in sleep deprivation [58], a state where mental fatigue is often prominent.

In states of anxiety and stress, often experienced as secondary to mental fatigue, increased glucocorticoid levels have been demonstrated. Interestingly, long-term increases in glucocorticoids have been demonstrated to result in the production of both TNF-α and IL-1β [59].

## Could mental fatigue be the consequence of a dysfunction in a specific brain region?

**Figure 1:**
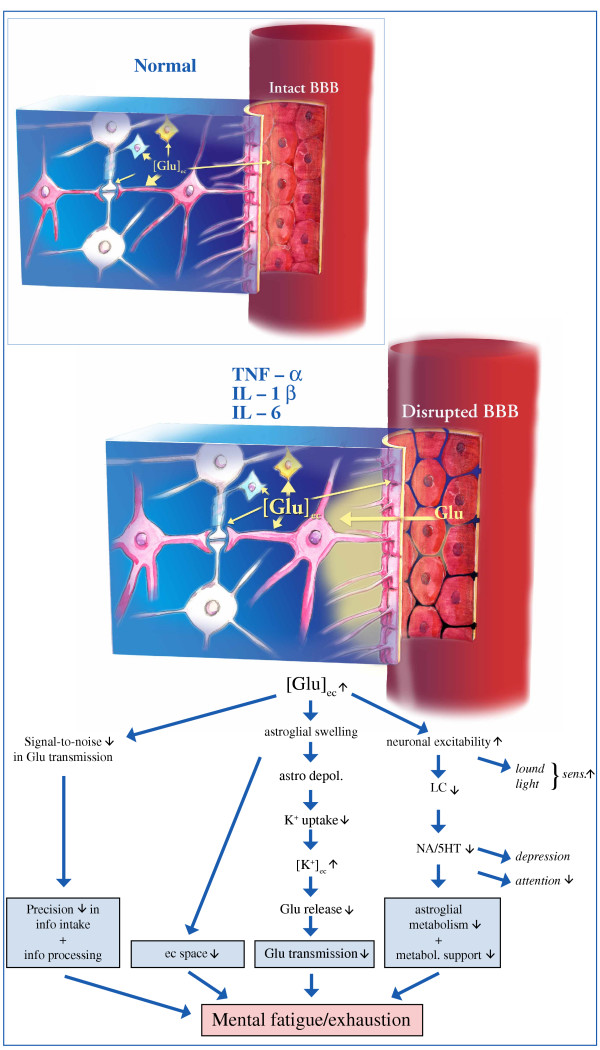
Schematic drawing of cellular regulation of extracellular glutamate concentrations ([Glu]_ec_) in normal brain function (left), and in the presence of the proinflammatory cytokines tumor necrosis factor-α (TNF-α), interleukin (IL)-1β, and IL-6 (right). Possible pathophysiology underlying mental fatigue at the cellular level is outlined below. To the left: Two neuronal cell bodies with processes (white) make contact with each other through a synapse (center). Astrocytic (pink) processes encapsulate the synapse and cover also the abluminal side of the blood vessel wall (right). The endothelial cells covering the luminal (blood) side of the vessel wall and the astrocytic processes make up the blood brain barrier (BBB). An oligodendroglial cell (bluish), with its myelin encapsulating the axon, and a microglial cell (yellow) are seen. The astrocytes, with their high-affinity glutamate transporters, are the main site for keeping [Glu]_ec _low. Even neurons express glutamate transporters, as do oligodendroglial cells, and endothelial cells at their abluminal side. To the right: TNF-α, IL-1β and IL-6 attenuate astroglial glutamate uptake transport and disintegrate the BBB, allowing glutamate from the blood to enter the brain. The overall result is slightly increased [Glu]_ec_. Tumor necrosis factor-alfa also decreases oligodendroglial cell glutamate uptake [78], while microglial glutamate uptake has been demonstrated to increase (Persson, M., Hansson, E., and Rönnbäck, L, to be published), though not to levels to compensate for the decreased astroglial glutamate uptake capacity. Due to increased [Glu]_ec_, astroglial swelling is shown. Below: Hypothetic cellular events underlying mental fatigue. Slightly increased [Glu]_ec _could make the glutamate neurotransmission less distinct (decrease the signal-to-noise ratio). At the cellular level, there would be astroglial swelling, which in turn would decrease the local extracellular (ec) volume and, as a consequence, lead to further increased [Glu]_ec_. Astroglial swelling also depolarizes the astroglial cell membrane, which further attenuates the electrogenic glutamate uptake and, in addition, the astroglial K^+ ^uptake capacity. As a consequence, even [K^+^]_ec _may rise. The increased [K^+^]_ec_, together with decreased glutamine production and reduced glucose uptake concomitant with the decreased glutamate uptake, could lead to decreased presynaptic glutamate release and thereby decreased glutamate transmission, which, according to our hypothesis, is one cellular correlate to mental fatigue/exhaustion. Increased extracellular glutamate levels in the prefrontal region could lead to inhibition of the brain stem nuclei locus coeruleus (LC) and raphe nuclei and thereby inhibit noradrenaline (NA) and serotonin (5-HT) release in the cerebral cortex resulting in decreased astroglial metabolism and neuronal metabolic supply. Increased neuronal excitability may be part of the loudness and light sensitivity often accompanying the mental fatigue. In addition, the decrease in noradrenaline and serotonin release might be part of decreased attention and the appearance of depression often accompanying the mental fatigue.

In the search for pathophysiological correlates to fatigue in MS, Roelcke and co-workers [60] demonstrated reduced glucose metabolism in the frontal cortex and basal ganglia in MS patients with fatigue. A hypotheses by Chaudhuri and Behan [6] also focused on basal ganglia as one part of the brain crucial for mental fatigue to appear. Using patients with chronic fatigue syndrome, which is not however exactly the same as mental fatigue, studies have revealed prefrontal and temporal cortices, anterior cingulate and cerebellum as regions possibly involved in fatigue [61]. Interestingly these later studies also pointed at a possible connection between glutamate transmission and fatigue. Even if the mental fatigue is not the central problem in attention deficit hyperactivity disorder (ADHD), some of the symptoms in this disorder is similar to the symptom complex associated with mental fatigue, and there is some support for glutamate being involved in the disorder and its treatment [62] and also, at least hypothetically, a deficient astroglial metabolism due to decreased noradrenaline and serotonin levels [63]. Until now there is no evidence for a specific brain region being affected in mental fatigue. On the contrary, it seems that mental fatigue could appear from disturbances of different neuronal systems. We will therefore present a hypothesis (figure 1) where the functional disturbance of mental fatigue at the cellular level is coupled to the fine-tuning of the glutamate neurotransmission.

## Mental fatigue – a stereotypical reaction upon brain function disturbance – a hypothesis focusing on impaired glutamate neurotransmission (figure 1)

It may be that mental fatigue is a stereotypical reaction to disturbance of "higher" brain functions. The brain, with its billions of specialized neurons and supporting glial cells, works as a "whole" organ. Every disturbance of brain homeostasis, no matter where the anatomical localization is, would therefore attenuate brain capacity for information processing and, as a consequence, information intake. One way to diminish information intake and processing at the cellular level would be to impair glutamate neurotransmission by attenuating the glial support and especially diminishing the astroglial capacity to clear [Glu]_ec_. The initial consequence would be slightly increased [Glu]_ec_, with less precision in glutamate transmission. This would disintegrate the "filter", which normally selects information and prevents it from reaching the cerebral cortex. We can take the sound from a low-frequency fan as an example. This sound is normally sorted out after hearing it for a while. If this sound is handled with less precision by auditory recognition systems, it will continually be recognized by brain centers as "new" information and be processed in the cerebral cortex as long as the sound is on. The "filter" that normally restrains already recognized information from reaching higher brain centers, has been "opened". From a physiological point of view, it seems appropriate that the individual, and not the brain at the synaptic level, should determine which information should reach, and be processed by, the cerebral cortex. The decreased attention, increased loudness and light sensitivity, and irritability could be physiological ways of avoiding overstimulation of higher cortical centers. In case the individuals cannot protect themselves from too much sensory stimulation, the filter's opening leads to overstimulation of the cerebral cortex. Here, the final shutdown of the glutamate transmission could be one mechanism underlying mental exhaustion (figure 1).

In line with these theoretical proposals, increased [Glu]_ec _has in fact been demonstrated in MS, meningitis, and encephalitis, Alzheimer's disease, ischemia and traumatic brain injury [64-69]. Furthermore, it has been shown in experimental studies that even extracellular K^+ ^is involved in the post-traumatic hyperexcitability, and a recent study has proposed that the larger extracellular K^+ ^increase evoked by neuronal activity is a consequence rather than the primary mechanism underlying post-traumatic hyperexcitability [70].

The theory also involves the possibility of a disturbed noradrenaline/serotonin turnover in the cerebral cortex due to a slight hyperexcitability in the frontal cortex. Interestingly, increased [Glu]_ec _in the prefrontal cortex has been reported by Bossuet and coworkers [67] in asymptomatic simian immunodeficiency virus (SIV)mac251-infected macaques without major brain involvement, being consistent with our theory at least in this set of animal experiments. If valid even in humans, a disturbed noradrenaline/serotonin turnover in the cerebral cortex could be coupled to the disturbed attention and depression often occurring in addition to the mental fatigue [see [71-73]].

## Testing of the hypothesis

It is not possible at present to ultimately prove whether or not the altered neuronal-glial interactions in glutamatergic transmission induced by proinflammatory cytokines could serve as a model to explain cellular mechanisms underlying mental fatigue. Brain imaging techniques able to determine and follow [Glu]_ec _and [K^+^]_ec _over time would be important to use in humans suffering from mental fatigue. Today, this is not possible for technical reasons. Instead, we must use experimental systems to learn about glial cell biology and neuron-glia-neuron signaling and interactions, and thus test specific parts of the hypothesis. Neuroactive substances produced by, or altered conditions related to, the production of proinflammatory cytokines could be evaluated with regard to their effects on astroglial support of glutamate transmission, and especially glutamate transport capacity. The role of the intact astroglial network in higher brain functions (cognition and behavior) could be studied in animal models. Effects of astroglial dysfunction with regard to glutamate transport capacity would be of special interest. Even clinical studies with different treatment strategies could be important in casting some light on the accuracy of the hypothesis. Of utmost importance in all such studies would be test batteries making it possible to objectify and even quantify the degree of mental fatigue.

## Why do the symptoms persist in some patients?

Normally, mental fatigue and the associated symptoms disappear when the brain dysfunction is over. In some patients, the symptoms persist. We have at present no explanation for this, but if our hypothesis is correct, there could be a genetic failure preventing astroglial glutamate transporters from upregulating. Another explanation for why the symptoms persist could be that the pathological stimulation by brain plasticity creates new neuronal networks [18,36].

## Aspects of treatment

Providing information about mental fatigue, its cause and the prognosis, is of utmost importance for breaking the vicious circle, which comes with the risk for secondary anxiety and depression. Furthermore, it is important for the patient to imagine and learn how much sensory stimulation they can tolerate prior to feeling too exhausted. Due to recent results on changes in cell signaling and neuronal plasticity [18,36], it may be important to identify the symptoms and treat them as early as possible to avoid formation of new and functionally disturbing neuronal circuits due to overstimulation of neuronal-glial units. If our hypothesis is correct, it may be possible to further improve the symptoms by suppressing the production of proinflammatory cytokines and, thereby, restoring the normal astroglial glutamate uptake. In this context, xanthine derivatives may be of use [74]. Another substance, worth considering, may be minocycline, a synthetic tetracycline derivative that has been shown to attenuate microglial activation and, consequently, the production of proinflammatory cytokines [75]. During recent years substances, which enhances glutamate uptake have been identified. Nicergoline [76], different growth factors including pituitary adenylate cyclase-activating polypeptide (PACAP) [77], some low molecular weight factors [23] as well as metabotropic glutamate agonists [47] have all been able to stimulate glutamate transport in experimental systems and could be of interest in the pharmacotherapy of mental fatigue. Interestingly, even AMPA receptor modulators have been demonstrated as cognitive enhancers [10].

## List of abbreviations used

ADHD attention deficit hyperactivity disorder

AMPA alpha-amino-3-hydroxy-5-methyl-4-isoxazolepropionate

ATP adenosine triphosphate

BBB blood brain barrier

Ca^2+ ^calcium

Ec extracellular

GLAST glutamate aspartate transporter

GLT-1 glutamate transporter-1

[Glu]_ec _extracellular glutamate concentration

5-HT 5-hydroxytryptamine

ICD-10 International Classification of Diseases, 10^th ^revision

IL-1/-6 interleukin-1/-6

K^+ ^potassium

[K^+^]_ec _extracellular potassium concentration

LC locus coeruleus

LTP long term potential

MS multiple sclerosis

Na^+ ^sodium

NA noradrenaline

NFκB nuclear transcription factor kappaB

NMDA N-methyl-D-aspartate

NO nitric oxide

PACAP pituitary adenylate cyclase-activating polypeptide

PI3K phosphatidylinositol-3-kinase

PKC protein kinase C

Siv mac simian immunodeficiency virus macaques

TNF-α tumor necrosis factor alpha

## Competing interests

The author(s) declare that they have no competing interests.

## Authors' contributions

Equal contributions by both authors.
